# Novel variants in *TNRC6B* cause global developmental delay with speech and behavioral abnormalities, short stature, low body weight, café‐au‐lait spots, and metabolic abnormality

**DOI:** 10.1002/mgg3.2408

**Published:** 2024-02-26

**Authors:** Qi Yang, Shan Ou, Xunzhao Zhou, Sheng Yi, Li Lin, Shang Yi, Shujie Zhang, Zailong Qin, Jingsi Luo

**Affiliations:** ^1^ Guangxi Key Laboratory of Birth Defects Research and Prevention, Guangxi Key Laboratory of Reproductive Health and Birth Defects Prevention Maternal and Child Health Hospital of Guangxi Zhuang Autonomous Region Nanning China; ^2^ Department of Genetic and Metabolic Central Laboratory Maternal and Child Health Hospital of Guangxi Zhuang Autonomous Region Nanning China; ^3^ Guangxi Clinical Research Center for Pediatric Diseases Maternal and Child Health Hospital of Guangxi Zhuang Autonomous Region Nanning China

**Keywords:** novel de novo variants, novel phenotype, *TNRC6B* gene, TNRC6B deficiency syndrome

## Abstract

**Background:**

TNRC6B deficiency syndrome, also known as global developmental delay with speech and behavioral abnormalities (MIM 619243), is a rare autosomal dominant genetic disease mainly characterized by facial dysmorphism, developmental delay/intellectual disability (DD/ID), speech and language delay, fine and motor delay, attention deficit and hyperactivity disorder (ADHD), and variable behavioral abnormalities. It is caused by heterozygous variant in the *TNRC6B* gene (NM_001162501.2, MIM 610740), which encodes the trinucleotide repeat‐containing adaptor 6B protein.

**Methods:**

In this study, two Chinese patients with TNRC6B deficiency syndrome were recruited, and genomic DNA extraction from peripheral blood leukocytes of these parents and their family members was extracted for whole‐exome sequencing and Sanger sequencing.

**Results:**

Here, we report two unrelated Chinese patients diagnosed with TNRC6B deficiency syndrome caused by novel de novo likely pathogenic or pathogenic *TNRC6B* variants c.335C>T (p.Pro112Leu) and c.1632delC (p.Leu546fs*63), which expands the genetic spectrum of TNRC6B deficiency syndrome. The clinical features of the patients were DD/ID, delayed speech, ADHD, behavioral abnormalities, short stature, low body weight, café‐au‐lait spots, metabolic abnormalities, and facial dysmorphism including coarse facial features, sparse hair, frontal bossing, hypertelorism, amblyopia, strabismus, and downslanted palpebral fissures, which expands the phenotype spectrum associated with TNRC6B deficiency syndrome.

**Conclusion:**

This study expands the genotypic and phenotypic spectrum of TNRC6B deficiency syndrome. Our findings indicate that patients with TNRC6B deficiency syndrome should be monitored for growth and metabolic problems and therapeutic strategies should be developed to address these problems. Our report also suggests the clinical diversity of TNRC6B deficiency syndrome.

## Graphical abstract

In this article, we investigate the pathogenesis of two unrelated Chinese children who exhibited developmental delay/intellectual disability, delayed speech, attention deficit and hyperactivity disorder, behavioral abnormalities, short stature, low body weight, café‐au‐lait spots, metabolic abnormalities, and facial dysmorphism including coarse facial features, sparse hair, frontal bossing, hypertelorism, amblyopia, strabismus, and downslanted palpebral fissures. Two novel de novo likely pathogenic or pathogenic TNRC6B variants c.335C>T (p.Pro112Leu) and c.1632delC (p.Leu546fs*63) were found in these patients. This study expands the genetic and phenotype spectrum of TNRC6B deficiency syndrome.

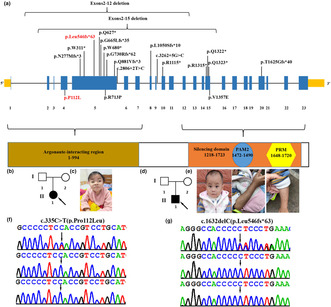

## 1 INTRODUCTION

**FIGURE 1:**
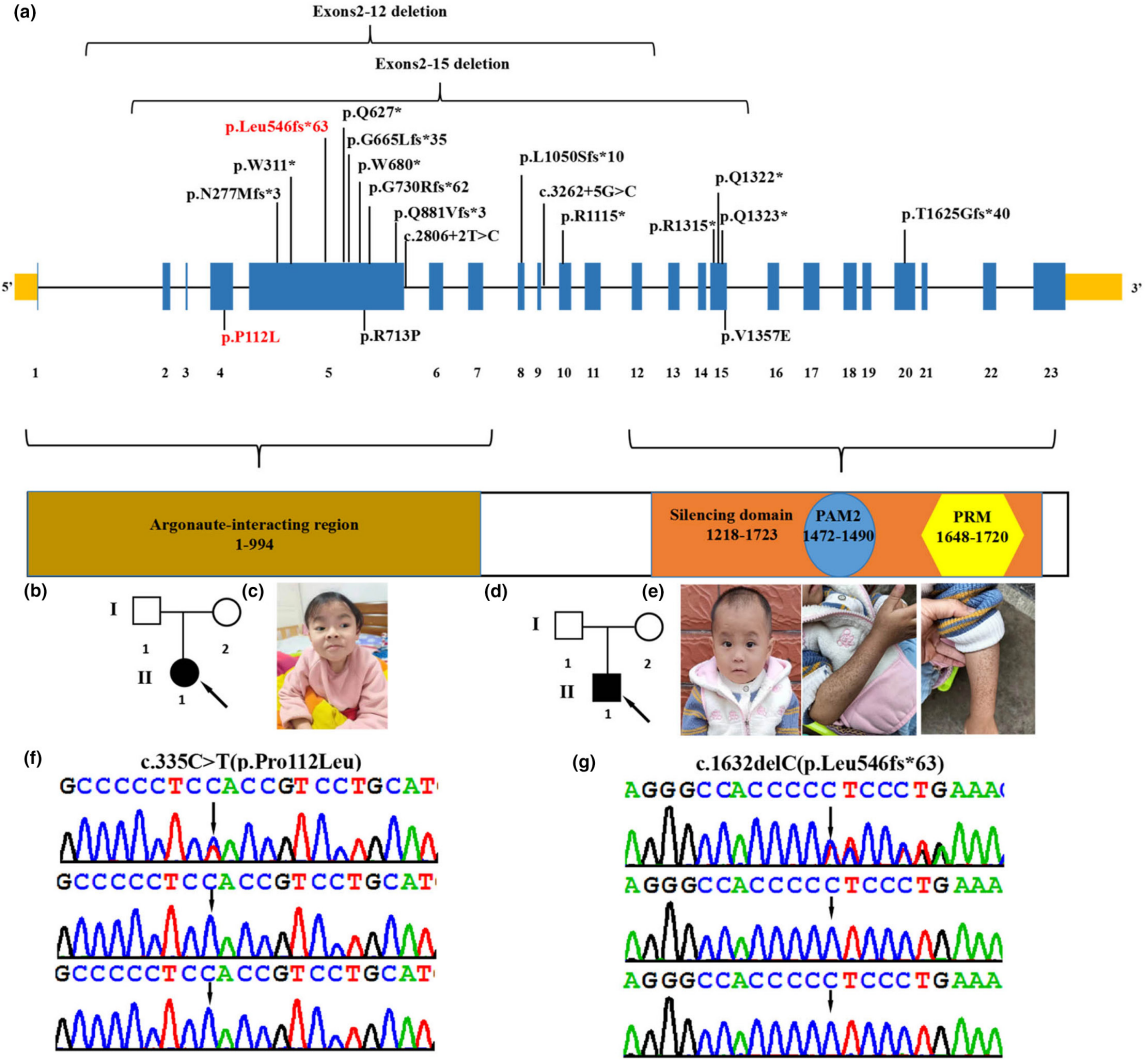
Clinical and genetic features. (a) The distribution of all TNRC6B variants detected so far in the 20 reported patients and 2 in this study. Boxes represent 23 different exons as indicated, and solid lines connecting these boxes represent the introns of *TNRC6B* gene (NM_001162501.2). The variants in our patients are highlighted in red. Schematic diagram of the location of the *TNRC6B* gene domains. (b) Pedigree chart of family 1 of the patient with TNRC6B deficiency syndrome. (c) Photograph of patient 1 (family 1, II‐1) at the age of 6 years and 6 months showing coarse facial features, sparse hair, hypertelorism, amblyopia, strabismus, and downslanted palpebral fissures. (d) Pedigree chart of family 2 of the patient with TNRC6B deficiency syndrome. (e) Photograph of patient 2 (family 2, II‐1) at the age of 2 years and 6 months showing sparse hair, frontal bossing, and hypertelorism. (f and g) Sanger sequencing of family 1 and family 2 with likely pathogenic variant.

TNRC6B deficiency syndrome, also known as global developmental delay with speech and behavioral abnormalities (GDSBA, MIM 619243), is caused by heterozygous variant in the *TNRC6B* (NM_001162501.2, MIM 610740) gene, located at 22q13.1, which contains 23 exons and encodes the trinucleotide repeat‐containing adaptor 6B protein involved in translational inhibition (Baillat & Shiekhattar, 2009; Granadillo et al., 2020). The TNRC6B protein comprises specific domains, including a GW repeat sequence essential for interaction with Argonaute proteins (Argonaute‐binding domain [ABD]), a glutamine‐rich region (Q‐rich) directing the localization of TNRC6B in the P‐bodies, an ubiquitin‐like‐associated structural domain, and a silencing structural domain (SD) responsible for mRNA‐binding repression (Baillat & Shiekhattar, 2009; Lazzaretti et al., 2009; Liu et al., 2018; Figure 1a). Functionally, TNRC6B mediates translational repression via miRNA‐guided mRNA cleavage and collaborates with Argonaute (Ago) family proteins in translational repression or mRNA degradation (Meister et al., 2005). The *TNRC6B* variant was first identified by whole‐exome sequencing (WES) in a large group of patients with autism without a detailed clinical description (Babbs et al., 2014; Iossifov et al., 2014). To date, only 19 variants in the *TNRC6B* gene have been reported in patients with TNRC6B deficiency syndrome (Bellido‐Cuéllar et al., 2023; Granadillo et al., 2020; Mitani et al., 2021). The 19 variants identified include 2 large exons deletion, 9 nonsense variants, 4 frameshift variants, 2 splicing variants, and 2 missense variants (Figure 1a). The clinical manifestations of TNRC6B deficiency syndrome are heterogeneous, with a wide range of symptoms including facial dysmorphism, development delay (DD)/intellectual disability (ID), speech and language delay, fine and motor delay, attention deficit and hyperactivity disorder (ADHD), autistic features, seizures, skeletal defects, and variable behavioral abnormalities (Granadillo et al., 2020). Due to the limited number of reported cases, a comprehensive clinical phenotype remains elusive, with only 21 reported patients to date. Here, we report two additional cases of TNRC6B deficiency syndrome with novel de novo heterozygous variants in the *TNRC6B* gene (NM_001162501.2; c.335C>T (p.Pro112Leu), c.1632delC (p.Leu546fs*63)). Furthermore, we provide additional molecular and clinical information for a better understanding of TNRC6B deficiency syndrome.

## 2 MATERIALS AND METHODS

### 2.1 Ethical compliance

This study was approved by the Institutional Review Board and Ethics Committee of Guangxi Maternal and Child Health Hospital (GXMC20230608). Written informed consent for genetic testing was obtained from the parents/guardians of the children.

### 2.2 Patient information

In this study, two children (one female, one male) from two unrelated Chinese families who presented with GDD/ID, speech and behavioral abnormalities, ADHD, short stature, low body weight, café‐au‐lait spots, metabolic abnormalities, and facial dysmorphism were recruited. We collected the peripheral blood leukocytes from the patients and their family members for WES and Sanger sequencing. We also collected and organized the hospitalization and examination data of these patients from the Maternal and Child Health Hospital of Guangxi Zhuang Autonomous Region.

### 2.3 Whole‐exome sequencing and sanger sequencing

Genomic DNA extraction from peripheral blood leukocytes of the parents and their family members was conducted using the Lab‐Aid DNA kit (Zeesan Biotech Co., Ltd., Xiamen, China). The DNA quality was measured with a NanoDrop2000 (Thermo Fisher Scientific, Waltham, MA, USA) and Qubit Fluorometer 3.0 (Thermo Fisher Scientific). WES was performed by Huada Gene Technology Co. Ltd (Shenzhen, China). Exons were captured using the Agilent SureSelect Human All ExonV5 Kit (Agilent, California, USA) based on the manufacturer's protocols. The enriched libraries underwent sequencing on a Hiseq2000 platform with 100 bp paired‐end reads. The Burrows–Wheeler aligner (BWA‐MEM, version 0.7.10) was used for reads aligned to the human genome assembly GRCh37. The Genome Analysis Toolkit 3.4 (GATK, www.broadinstitute.org/gatk) was then used to detect variants such as single‐nucleotide variants small insertions and deletions. Variants were annotated by TGEX software, with a minor allele frequency below 0.5% according to either the public databases (e.g., 1000 Genomes Project, Exome Sequencing Project, and ExAC) or our in‐house databases. In silico tools Mutation taster (http://www.mutationtaster.Org; disease‐causing, polymorphism), Polyphen2 (http://genetics.bwh.harvard.edu/pph2/; 0–0.15 Benign, 0.15–0.85 Possibly damaging, 0.85–1 Probably damaging), Sorts Intolerant From Tolerant (SIFT; http://sift.bii.a‐star.edu.sg/www/SIFT_seq_submit2.html; <0.05 deleterious, ≥0.05 tolerated), and Combined Annotation Dependent Depletion (CADD; https://cadd.gs.washington.edu/snv; >20 Damaging, ≤2 Tolerable) were used to analyze the pathogenicity of the variants. The pathogenicity of identified variants was interpreted and classified according to the American College of Medical Genetics (ACMG)/Association of Molecular Pathology (AMP) guidelines (Richards et al., 2015).

## 3 RESULT

### 3.1 Clinical features

Patient 1 was a female first seen at the Department of Pediatrics, Maternal and Child Health Hospital of Guangxi Zhuang Autonomous Region at the age of 6 years and 6 months for DD/ID (Figure 1b; Table 1). She was born by cesarean section at the gestational age of 39 + 3 weeks with birth weight (3150 g) and birth length (47.8 cm). Her development was mildly delayed as she started walking at 17 months independently. She began to speak several words at 2 years old. A physical examination showed that she had a moderately short stature with a body length of 109.2 cm (<2 SD). She was diagnosed with ADHD as well as irritability. She had learning difficulties with slowed information processing, weak memory, and poor concentration. At the age of 6 years and 6 months, she underwent a cognitive level test using the Wechsler Intelligence Scale for Children: 4th edition (WISC‐IV), which revealed that her full‐scale IQ was 51, Verbal Comprehension Index was 65, Perceptual Reasoning Index was 71, Working Memory Index was 65, and Processing Speed Index was 64. At 6 years and 7 months of age, brain magnetic resonance imaging performed revealed widening extracerebral spaces at the left temporal lobes. Electroencephalography, performed 6 years and 6 months, showed bilateral mild diffuse slow waves. Her facial dysmorphism included coarse facial features, sparse hair, hypertelorism, amblyopia, strabismus, and downslanted palpebral fissures (Figure 1c). The patient's x‐ray of the chest was normal.

**TABLE 1:** Clinical features of the patient with de novo *TNRC6B* mutations.

Clinical features	Patient 1	Patient 2
Variants in TNRC6B (NM_001162501.2)	c.335C>T (p.Pro112Leu)	c.1632delC (p.Leu546fs*63)
Gender	Female	Male
Age at last examination	6 years and 7 months	2 years and 7 months
Gestation	Full‐term	Full‐term
Muscular hypotonia	Yes	Normal
Weight	22.4 kg	9.7 kg (≤3SD)
Height	109.2 cm (≤2SD)	80 cm (≤3SD)
Developmental delay/movement delay/intellectual disability	Yes; mild	Yes; mild
IQ	51	96
Age of walking	17 months	16 months
Age of first words	24 months	20 months
Behavior anomalies	Attention deficit–hyperactivity disorder (ADHD), irritability, slowed information processing, weak memory, and poor concentration	Normal
Brain anomalies	Widening extracerebral spaces at the left temporal lobes	MRI normal
Unusual hair	Sparse hair	Sparse hair
Facial dysmorphisms	Coarse facial features, hypertelorism, amblyopia, strabismus, and downslanted palpebral fissures	Frontal bossing and hypertelorism
Electroencephalogram	Bilateral mild diffuse slow waves	Normal
Other anomalies	No	Café‐au‐lait spots and metabolic abnormalities

Patient 2, a male child, was admitted to the Department of Pediatrics of Guangxi Zhuang Autonomous Region Maternal and Child Health Hospital at the age of 2 years and 6 months for recurrent diarrhea and vomiting (Figure 1d; Table 1). Physical examinations revealed that he had severe short stature (80 cm, ≤3 SD), low body weight (weight ≤3 SD), and craniofacial deformities, including frontal bossing, sparse hair, and hypertelorism (Figure 1e). He had multiple pigmented skin spots on his hands and chest that appeared to be Cafe‐au‐Lait spots (Figure 1e). His speech was moderately delayed as he began to speak several words at 20 months. His motor was mildly delayed as he started walking at 16 months independently. His intellectual development is normal (IQ: 96). The initial laboratory results showed electrolyte disturbances (Na^+^: 149.0 mmol/L, normal 135–145 mmol/L. K^+^: 3.9 mmol/L, normal 4.1–5.3 mmol/L), metabolic acidosis (pH = 7.13, pCO_2_ = 22.0 mmHg, PO_2_ = 114.0 mmHg, HCO_3_ = 7.3 mmol/L, lactate =4.9 mmol/L), hyperammonemia (73.0 μmol/L), hypoglycemia (3.10 mmol/L), hypoinsulinemia (1.7 pmol/L), hyperbilirubinemia (total bilirubin: 23.74 μmol/L), hypoproteinemia (total protein: 45.27 g/L, albumin: 29.93 g/L), hypocholesterolemia (total cholesterol: 1.70 mmol/L), and elevated liver enzymes (AST 638 U/L and ALT 1163 U/L). Blood tandem mass spectrometry revealed a decrease in citrulline (1.75 μmol/L, normal 5.5–41.00 μmol/L) and tyrosine (32.22 μmol/L, normal 45.00–260.00 μmol/L). The endocrine evaluations showed laboratory signs of adrenocortical dysplasia: plasma aldosterone 213.66 pg/mL, midnight cortisol 65.43 nmol/L, midnight adrenocorticotropic hormone (ACTH) 2.01 pmol/L, morning cortisol 212.98 nmol/L, and morning ACTH 7.35 pmol/L. He had been admitted to the hospital several times previously because of infections, vomiting, diarrhea, and metabolic acidosis. The patient was suspected of Woolman's syndrome and was treated with hydrocortisone acetate in doses of 1.6 mg tid. The patient's x‐ray of the chest and spine, and abdominal and adrenal gland ultrasound examinations were normal.

### 3.2 Molecular analysis

WES identified two heterozygous variants in the *TNRC6B* gene (NM_001162501.2) in two patients: c.335C>T (p.Pro112Leu) and c.1632delC (p.Leu546fs*63), respectively (Figure 1f,g). These variants were not identified in patients' parents by Sanger sequencing, suggesting that they were de novo variants. These variants were confirmed to be novel variants absent from the Human Gene Mutation database, Exome Sequencing Project, gnomAD, ClinVar, 1000 Genomes Project, and the Single Nucleotide Polymorphism database. The two novel de novo variants are predicted to be deleterious by in silico tools. The pathogenicity prediction analysis and ACMG/AMP rating of the two *TNRC6B* variants are shown in Table 2.

**TABLE 2:** Predicted pathogenicity of de novo *TNRC6B* variants.

Patient	Variant (NM_001162501.2)	Inheritance	Mutation taster	PolyPhen‐2	SIFT	CADD	ACMG/AMP
Patient 1	c.335C>T (p.Pro112Leu)	DNV	D	D (1.000)	PD (0.113)	28.9	P (PS2+PM2+PP3)
Patient 2	c.1632delC (p.Leu546fs*63)	DNV	D	N.A.	N.A.	N.A.	P (PVS1+PS2+PM2)

Abbreviations: ACMG/AMP, American College of Medical Genetics/Association of Molecular Pathology; CADD, Combined Annotation Dependent Depletion; D, deleterious or damaging; DNV, de novo variant; N.A. not available; P, pathogenic; PD, probably damaging; SIFT, Sorts Intolerant From Tolerant.

## 4 DISCUSSION

TNRC6B‐deficiency syndrome is a rare disease characterized by facial dysmorphism, DD/ID, speech and language delay, fine and motor delay, ADHD, and variable behavioral abnormalities (Granadillo et al., 2020). The *TNRC6B* variant was first identified by WES in a large group of patients with autism without a detailed clinical description (Babbs et al., 2014; Iossifov et al., 2014). Then, Granadillo et al. (2020) described in detail the clinical phenotype and molecular genetic features of 17 patients with TNRC6B‐related disease. Bellido‐Cuéllar et al. (2023) further expanded the clinical phenotype of the TNRC6B deficiency syndrome by studying *TNRC6B* variant in a case with ASD and generalized epilepsy. To date, only 20 individuals with global developmental delay with speech and behavioral abnormalities have been reported (Babbs et al., 2014; Bellido‐Cuéllar et al., 2023; Granadillo et al., 2020; Iossifov et al., 2014; Mitani et al., 2021). Here, we reported two additional Chinese patients of global developmental delay with speech and behavioral abnormalities caused by two novel de novo, heterozygous variants in the *TNRC6B* gene, c.335C>T (p.Pro112Leu) and c.1632delC (p.Leu546fs*63), respectively.

Clinically, our patients were similar to those of the reported cases that included DD/ID, facial dysmorphism, delayed speech, ADHD, and behavioral abnormalities. However, our patients had several additional features, further expanding the TNRC6B deficiency syndrome‐related phenotype. Postnatal growth indicators were within the normal range in most patients. Of note, both of our patients exhibited moderate‐to‐severe short stature. We also noted that patient 2 had a low body weight. We also investigated the possibility of linear growth abnormalities in other patients with TNRC6B deficiency syndrome (Babbs et al., 2014; Bellido‐Cuéllar et al., 2023; Granadillo et al., 2020; Iossifov et al., 2014; Mitani et al., 2021). Mitani et al. (2021) reported that two siblings with homozygous pathogenic variants of TNRC6B had severe short stature and low body weight (Liu et al., 2018). We also noticed in Granadillo et al. (2020) report that there was one patient with a weight below 2SD. Therefore, patients with TNRC6B deficiency syndrome should be monitored for growth problems.

Although dysmorphic facial features are a common clinical phenotype of the syndrome, we did not find a common facial profile. To the best of our knowledge, sparse hair and hypertelorism in patients 1 and/or 2 have not been described in reported cases. In addition, patient 2 also had numerous café‐au‐lait spots on his hands and chest. This skin pigmentation phenotype has never been observed before in a case of TNRC6B‐related disease.

Patient 1 harbors a missense variant, while patient 2 has a null variant. However, we observed more severe developmental abnormalities in patient 2. Similarly, in Granadillo et al. (2020) study, patients carrying missense variants also exhibited more serious developmental issues. Additionally, Mitani et al. (2021) reported that two patients with missense variants also exhibited severe developmental abnormalities (Liu et al., 2018). Although some patients with null variants also showed serious developmental problems, it appears that patients carrying missense mutations may be more prone to severe developmental issues. Considering the limitations of the cases and variants reported to date, these results should be considered provisional. Therefore, it is essential for future studies to investigate a larger cohort of patients to refine the phenotype, elucidate genotype effects, and identify other phenotype‐determining factors.

Patient 2 was considered to have Wolman disease at the age of 2 years and 5 months based on adrenocortical dysplasia and abnormal liver function. Wolman's disease is a severe metabolic disorder characterized by absent or very low lysosomal acid lipase activity and cholesteryl ester accumulation, associated with hepatosplenomegaly and adrenal calcification, and is usually fatal within the first year of life. It is caused by variants in the *LIPA* gene. However, whole‐exome analysis did not reveal any variants in the *LIPA* gene nor did it reveal any variants in other genes associated with adrenocortical hypoplasia (e.g., *NR0B1*, *NR5A1*). Furthermore, patient 2 exhibited other symptoms such as liver function abnormalities and café‐au‐lait spots; however, WES analysis did not reveal any other gene variants associated with these symptoms in the patient. Similarly, in Granadillo et al. (2020) study, a patient with a *TNRC6B* variant had short‐chain acyl‐CoA dehydrogenase deficiency, and genetic testing revealed no variants associated with short‐chain acyl‐CoA dehydrogenase deficiency. Despite these two patients carrying the same type of variation, they exhibit different phenotypes. Although both patients have metabolic abnormalities, our patient presents with adrenocortical dysplasia, while the patient reported by Granadillo et al. (2020) has short‐chain acyl‐CoA dehydrogenase deficiency. Additionally, both patients exhibit developmental delay, but the patient in Granadillo et al. (2020) study shows a more severe presentation. Furthermore, our patient also manifests additional phenotypes such as liver function abnormalities and café‐au‐lait spots. These results further underscore the complexity and heterogeneity of the phenotype in TNRC6B deficiency syndrome. Phenotypic variations observed in these patients may be attributed to functional loss mutations occurring at different positions, potentially exerting site‐specific effects on the phenotype. Although genes involved in RNA regulation (such as *TNRC6B*) are not associated or are weakly associated with metabolic abnormalities, liver function abnormalities, and café‐au‐lait spots, considering that *TNRC6B* is expressed in all organs of the body, such as kidney, liver, and gastrointestinal tract, it cannot be ruled out that it may affect the development of the corresponding organs, leading to metabolic abnormalities. To assess the significance of these differences, additional patients and functional studies of *TNRC6B* variants are needed.

**FIGURE 2:**
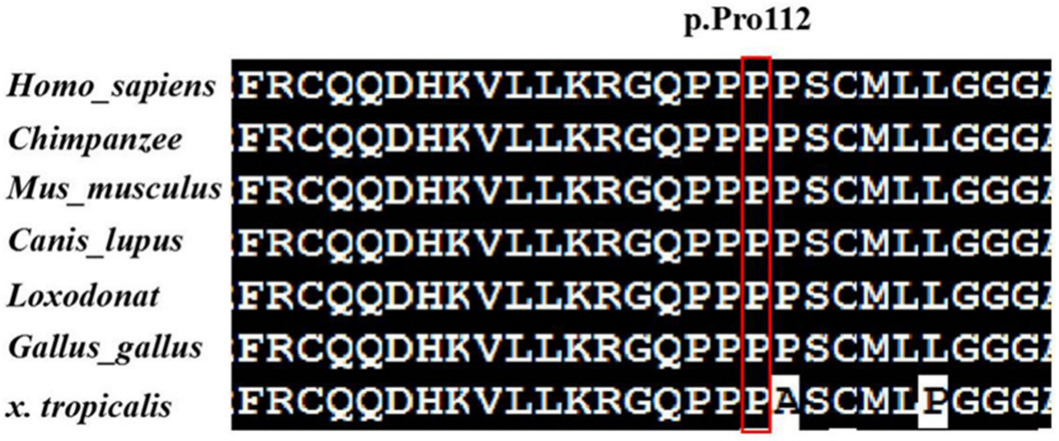
Multispecies alignment showing the strong conservation of TNRC6B p.Pro112.

The variant c.335C>T (p.Pro112Leu) was predicted to be deleterious by in silico prediction tools (e.g., SIFT, PolyPhen2, CADD, and MutationTaster). In Figure 2, multiple‐sequence alignments showed that the sequence at residue 112 is highly conserved among different organisms. The variant c.335C>T (p.Pro112Leu) is located in the fourth exon of the *TNRC6B* gene and in the N‐terminal of the TNRC6B protein, which is necessary for the interaction with Argonaute proteins (ABD). The other *TNRC6B* variant c.1632delC (p.Leu546fs*63) is located in the fifth exon of the *TNRC6B* gene and causes a frameshift alteration after codon 546 leading to a premature termination codon (PTC) which is located at codon 609, resulting in truncation of the TNRC6B protein that undergoes nonsense‐mediated decay (NMD). According to the ACMG/AMP standards and guidelines, c.335C>T (p.Pro112Leu) was classified to be likely pathogenic (PS2, PM2, PP3), and c.1632delC/p.Leu546fs*63 was classified to be pathogenic (PVS1, PS2, PM2). TNRC6B is expressed in all tissues of the body, including high expression in the frontal cortex, basal ganglia, and cerebellum. This may indicate an underlying mechanism for DD/ID and behavioral abnormalities in most cases, as well as a possible mechanism for the patient's inconsistent congenital malformations. Future in vivo and in vitro experiments are needed to explore the pathogenic mechanisms by which TNRC6B mutations cause multisystem abnormalities.

## 5 CONCLUSION

In conclusion, we described two unrelated Chinese patients of global developmental delay with speech and behavioral abnormalities with novel de novo *TNRC6B* variants in detail. Clinical features of the patients included DD/ID, delayed speech, ADHD and behavioral abnormalities, and facial dysmorphism including coarse facial features, sparse hair, frontal bossing, hypertelorism, amblyopia, strabismus, and downslanted palpebral fissures. In addition, these patients exhibit short stature, and patient 2 also has a low body weight, café‐au‐lait spots, and metabolic abnormalities. Our report expands the phenotype and genetics spectrum of TNRC6B‐related disease.

## AUTHOR CONTRIBUTIONS

QY and JSL contributed to design and writing of the study. SO, XZZ, SheY, LL, and ShaY collected and analyzed the WES data. SO, SJZ, and ZLQ modified the manuscript. All authors read and approved the final version of the manuscript.

## FUNDING INFORMATION

This research was supported by the Health Department of Guangxi Province (Grant No. Z‐A20220256 and Z‐A20220268), the Guangxi Medical and Health Appropriate Technology Development and Promotion Application (S2020060), and the Guangxi Clinical Research Center for Pediatric Diseases (Guike AD22035121).

## CONFLICT OF INTEREST STATEMENT

The authors declare that the research was conducted in the absence of any commercial or financial relationships that could be construed as a potential conflict of interest.

## ETHICS STATEMENT

The study was approved by the Institutional Review Board and Ethics Committee of Guangxi Maternal and Child Health Hospital (GXMC20230608). The study was conducted in accordance with the Good Clinical Practice and the Declaration of Helsinki. Detailed written informed consent for publishing data and images was obtained from all participants or legal guardians included in this study.

## Data Availability

All data that support the findings of the current study are available from the corresponding author upon reasonable request.

## References

[mgg32408-bib-0001] Babbs, C. , Lloyd, D. , Pagnamenta, A. T. , Twigg, S. R. , Green, J. , McGowan, S. J. , Mirza, G. , Naples, R. , Sharma, V. P. , Volpi, E. V. , Buckle, V. J. , Wall, S. A. , Knight, S. J. , International Molecular Genetic Study of Autism Consortium (IMGSAC) , Parr, J. R. , & Wilkie, A. O. (2014). De novo and rare inherited mutations implicate the transcriptional coregulator TCF20/SPBP in autism spectrum disorder. Journal of Medical Genetics, 51(11), 737–747. 10.1136/jmedgenet-2014-102582 25228304 PMC4215269

[mgg32408-bib-0002] Baillat, D. , & Shiekhattar, R. (2009). Functional dissection of the human TNRC6 (GW182‐related) family of proteins. Molecular and Cellular Biology, 29(15), 4144–4155. 10.1128/MCB.00380-09 19470757 PMC2715800

[mgg32408-bib-0003] Bellido‐Cuéllar, S. , Pérez de la Fuente, R. , Lezana‐Rosales, J. M. , Sánchez‐Calvín, M. T. , Saiz‐Díaz, R. A. , & González de la Aleja, J. (2023). Epilepsy and autism spectrum disorder caused by a pathogenic variant in TNRC6B. Seizure, 110, 117–118. 10.1016/j.seizure 37348364

[mgg32408-bib-0004] Granadillo, J. L. , P A Stegmann, A. , Xia, K. , Angle, B. , Bontempo, K. , Ranells, J. D. , Newkirk, P. , Costin, C. , Viront, J. , Stumpel, C. T. , Sinnema, M. , Panis, B. , Pfundt, R. , Krapels, I. P. C. , Klaassens, M. , Nicolai, J. , Li, J. , Jiang, Y. , Marco, E. , … Shinawi, M. (2020). Pathogenic variants in TNRC6B cause a genetic disorder characterised by developmental delay/intellectual disability and a spectrum of neurobehavioural phenotypes including autism and ADHD. Journal of Medical Genetics, 57(10), 717–724. 10.1136/jmedgenet-2019-106470 32152250

[mgg32408-bib-0005] Iossifov, I. , O'Roak, B. J. , Sanders, S. J. , Ronemus, M. , Krumm, N. , Levy, D. , Stessman, H. A. , Witherspoon, K. T. , Vives, L. , Patterson, K. E. , Smith, J. D. , Paeper, B. , Nickerson, D. A. , Dea, J. , Dong, S. , Gonzalez, L. E. , Mandell, J. D. , Mane, S. M. , Murtha, M. T. , … Wigler, M. (2014). The contribution of de novo coding mutations to autism spectrum disorder. Nature, 515(7526), 216–221. 10.1038/nature13908 25363768 PMC4313871

[mgg32408-bib-0006] Lazzaretti, D. , Tournier, I. , & Izaurralde, E. (2009). The C‐terminal domains of human TNRC6A, TNRC6B, and TNRC6C silence bound transcripts independently of Argonaute proteins. RNA (New York, N.Y.), 15(6), 1059–1066. 10.1261/rna.1606309 19383768 PMC2685519

[mgg32408-bib-0007] Liu, J. , Liu, Z. , & Corey, D. R. (2018). The requirement for GW182 scaffolding protein depends on whether Argonaute is mediating translation, transcription, or splicing. Biochemistry, 57(35), 5247–5256. 10.1021/acs.biochem.8b00602 30086238 PMC6124307

[mgg32408-bib-0008] Meister, G. , Landthaler, M. , Peters, L. , Chen, P. Y. , Urlaub, H. , Lührmann, R. , & Tuschl, T. (2005). Identification of novel argonaute‐associated proteins. Current Biology: CB, 15(23), 2149–2155. 10.1016/j.cub.2005.10.048 16289642

[mgg32408-bib-0009] Mitani, T. , Isikay, S. , Gezdirici, A. , Gulec, E. Y. , Punetha, J. , Fatih, J. M. , Herman, I. , Akay, G. , du, H. , Calame, D. G. , Ayaz, A. , Tos, T. , Yesil, G. , Aydin, H. , Geckinli, B. , Elcioglu, N. , Candan, S. , Sezer, O. , Erdem, H. B. , … Pehlivan, D. (2021). High prevalence of multilocus pathogenic variation in neurodevelopmental disorders in the Turkish population. American Journal of Human Genetics, 108(10), 1981–2005. 10.1016/j.ajhg.2021.08.009 34582790 PMC8546040

[mgg32408-bib-0010] Richards, S. , Aziz, N. , Bale, S. , Bick, D. , Das, S. , Gastier‐Foster, J. , Grody, W. W. , Hegde, M. , Lyon, E. , Spector, E. , Voelkerding, K. , Rehm, H. L. , & ACMG Laboratory Quality Assurance Committee . (2015). Standards and guidelines for the interpretation of sequence variants: a joint consensus recommendation of the American College of Medical Genetics and Genomics and the Association for Molecular Pathology. Genetics in Medicine: Official Journal of the American College of Medical Genetics, 17, 405–424. 25741868 10.1038/gim.2015.30 PMC4544753

